# The Obesity-Breast Cancer Conundrum: An Analysis of the Issues

**DOI:** 10.3390/ijms17060989

**Published:** 2016-06-22

**Authors:** Shawna B. Matthews, Henry J. Thompson

**Affiliations:** Cancer Prevention Laboratory, Colorado State University, Fort Collins, CO 80523, USA; shawna.matthews@ucdenver.edu

**Keywords:** breast cancer, obesity, mechanisms, preclinical models

## Abstract

Breast cancer develops over a timeframe of 2–3 decades prior to clinical detection. Given this prolonged latency, it is somewhat unexpected from a biological perspective that obesity has no effect or reduces the risk for breast cancer in premenopausal women yet increases the risk for breast cancer in postmenopausal women. This conundrum is particularly striking in light of the generally negative effects of obesity on breast cancer outcomes, including larger tumor size at diagnosis and poorer prognosis in both pre- and postmenopausal women. This review and analysis identifies factors that may contribute to this apparent conundrum, issues that merit further investigation, and characteristics of preclinical models for breast cancer and obesity that should be considered if animal models are used to deconstruct the conundrum.

## 1. Overview

In 2012, 1.7 million new cases of breast cancer were diagnosed globally, and breast cancer was responsible for nearly 700,000 deaths. Worldwide, cumulative lifetime risk for women of developing breast cancer between the ages of 0 and 74 currently stands at 4.6%, and lifetime risk of dying from breast cancer is 1.4% [1]. In the United States, one in eight women (12.5%) will be diagnosed with breast cancer during her lifetime. As both the most commonly detected and second leading cause of cancer death in women in the United States, breast cancer is diagnosed every two minutes, and breast cancer kills a woman every eleven minutes [2].

The American Cancer Society divides breast cancer risk factors into two categories based on whether they are related to lifestyle [2]. Relative to factors unrelated to lifestyle, seven breast cancer susceptibility genes or gene sets with high penetrance (relative risk > 5) have been identified in which germline mutations are associated with increased breast cancer risk in an inherited Mendelian fashion [3]. These genes are: DNA damage repair genes *BRCA1/2*, cell–cell adhesion gene *CDH1*, tumor suppressor phosphatase and tensin homolog *PTEN*, serine threonine kinase *SKT11*, tumor suppressor *TP53*, and DNA mismatch repair genes *MLH1*, *MSH2/6*, and *PMS2* [3]. These seven genes are cumulatively found to be drivers in only 5%–10% of all breast cancer cases, leaving the majority of breast cancer cases attributable to a complex summation of genetic and epigenetic alterations on which personal choices, generally categorized as environmental exposures, are thought to operate.

## 2. Breast Cancer and Obesity: What Is the Connection?

Of the risk factors related to personal choice, chronic positive energy balance, manifested as excess adiposity, has been linked to the development of cancer [4,5,6]. Body mass index (BMI, weight (kg)/height (m^2^)) is frequently used as a proxy for body fatness; a BMI ≥ 25–29.9 kg/m^2^ is considered overweight, and BMI ≥ 30 kg/m^2^ is considered obese [7,8]. Breast cancer incidence in Western countries, including the United States, has risen by over 30% in the past 25 years [2]. While attributed in part to changes in reproductive patterns and improved detection methods, this increased breast cancer incidence may also reflect the rising prevalence of hallmarks of Western culture such as obesity and physical inactivity [2]. Howell *et al.* concluded that lifestyle modifications, including maintaining or returning to BMI < 25, engaging in moderate physical activity, and limiting alcohol to <3 drinks per week, can cumulatively reduce breast cancer risk by >30% [9]. Of these factors, the World Cancer Research Fund estimates that 17% of breast cancer diagnoses in the United States could be prevented by maintaining a healthy weight [10]. This relationship is of considerable public health importance given the ongoing obesity epidemic, in which two out of three women in the United States are overweight or obese with BMI ≥ 25.

## 3. Menopausal Status, Obesity, and Breast Cancer

Menopausal status is the fulcrum on which the association between obesity and breast cancer has been reported to hinge. However, inconsistencies in the data suggest that the complexity of the relationship between obesity and breast cancer remains inadequately understood. The inconsistencies exist at the level: (1) of population data for incident disease risk; (2) of clinical data on the characteristics of the disease at diagnosis and on disease prognosis and outcomes; and (3) of the four mechanisms broadly cited to explain the obesity-breast cancer linkage, *i.e.*, (a) sex hormone metabolism; (b) deregulated insulin signaling; (c) chronic low grade inflammation; and (d) altered adipokine expression.

### 3.1. Premenopausal Breast Cancer

Most studies report a null or inverse association of obesity with breast cancer risk in premenopausal women (relative risk (RR) = 0.92 (95% CI, 0.88–0.97), *p* = 0.001; approximately 10% reduced risk per 5 kg per m^2^) [10,11,12,13,14]. This effect appears to be primarily on tumors that express the estrogen receptor (ER) and/or progesterone receptor (PR); in contrast, risk for ER/PR negative and triple negative breast cancers is increased (80% increased risk per 5 kg per m^2^) [15]. While there is general consensus about the inverse relationship between excess body fat and breast cancer risk in premenopausal women, there are notable exceptions. For example, in premenopausal women at high risk for breast cancer as defined by the Gail score, risk of invasive breast cancer was significantly increased in overweight (hazard ratio (HR) = 1.59 (1.05–2.42)) and obese (HR = 1.70 (1.10–2.63), *p*_trend_ = 0.01) women compared to women of BMI < 25 [16]. While regular mammographic screening (and hence earlier detection) of the cohorts reported in this study may account for the positive association observed, biological mechanisms may exist as well. Breast palpation and detection of lumps is more difficult in obese women compared to other women [17]. Consequently, heavier women are more likely to have a delayed diagnosis compared to women of normal weight. This could delay the detection of breast cancer into the postmenopausal stage of life despite the tumor existing before menopause, causing the association to appear stronger among postmenopausal women.

Other notable exceptions to the obesity and premenopausal breast cancer risk paradigm comes from studies of Asia-Pacific women, in which increased BMI is associated with an increased risk of premenopausal breast cancer (approximately 15% increased risk per 5 kg per m^2^) [14,18]. Likewise, investigations into the relationship between obesity and breast cancer may be influenced by use of body mass index (BMI) as an indirect estimate of body fatness, as opposed to direct measures of adiposity. Evidence in support of this theory comes from studies of women from North American and Europe, in which associations between anthropometric measures of abdominal fatness such as waist circumference and waist/hip ratio, which are generally considered more indicative of adiposity, and premenopausal breast cancer risk are generally null (not protective) or modestly positive (slightly increase risk) [19,20,21]. Importantly, the majority (>90%) of these women were actively cycling. Thus, the widely accepted convention that there is an inverse association between premenopausal breast cancer risk and obesity not only has notable exceptions, but these exceptions are not readily explained by the commonly proposed mechanism that ovarian-derived androgen excess and chronic anovulation, resulting in reduction of luteal-phase progesterone production (progesterone deficiency), underlie the inverse association [22,23].

### 3.2. Postmenopausal Breast Cancer

In postmenopausal women, the majority of evidence supports a direct link between obesity and postmenopausal breast cancer risk (relative risk (RR) = 1.12 (95% CI, 1.09–1.16), *p* < 0.001; a 12%–13% increase in risk per 5 kg per m^2^) [6,10,14]. While weaker in magnitude, increased breast cancer risk was also reported in overweight and obese postmenopausal women with a Gail score greater than 1.67 (overweight, HR = 1.07 (95% CI, 0.88–1.30); obese, HR = 1.14 (95% CI, 0.94–1.38), compared to women of BMI < 25 (*p*_trend_ = 0.17) [16]. Whether this reflects true differences in the biology of the obesity-breast cancer relationship in women considered high-risk by Gail score versus those who are not considered high-risk, or whether this finding is due to the study design, which included regular mammographic screening and clinically measured *versus* self reported BMI (as is typical in most population based studies), is unclear [13,16].

When limited to ER/PR positive breast cancer, a 33% risk increase in postmenopausal women is estimated per 5 kg per m^2^ BMI increment [24]. The predominant hypothesis invoked to explain the increased risk conferred by obesity centers on peripheral production of sex hormones by fat tissue; specifically, the higher rates of conversion of androgenic precursors to estradiol through increased aromatase enzyme activity, a process known as aromatization [14]. This hypothesis predicts the strongest associations with ER/PR positive (luminal subtypes) of breast cancer.

For molecular subtypes other than luminal breast tumors, results have been mixed. While most cohort studies find no association [25,26,27,28,29] or a reduced risk with increased BMI [30], positive associations between increasing BMI and the incidence of triple-negative tumors have been reported [31,32]. Collectively, these findings indicate that additional insights require thinking beyond the boundaries currently defined by the peripheral aromatization hypothesis despite population derived evidence that obesity associated breast cancer risk in postmenopausal women is explained almost entirely explained by the increase in estradiol levels with higher BMI [33].

## 4. Obesity Negatively Influences Breast Cancer Outcomes

Unlike the situation described above, the effect of obesity on breast cancer outcomes is independent of menopausal status. Obese women with breast cancer typically have larger tumors, advanced disease stage at diagnosis, higher rates of metastasis, and higher rates of distant recurrence (HR = 1.57 (95% CI, 1.11–2.22)) [34,35,36,37,38]. Obese women with breast cancer have increased chance of initial or acquired chemotherapy resistance, and obese women have higher all-cause mortality (HR = 1.56 (95% CI, 1.01–2.40)) and breast cancer-related mortality (HR = 2.54 (95% CI, 1.08–6.00)) at any age compared to normal weight women with breast cancer. In a cohort of *N* = 462 premenopausal women and *N* = 972 postmenopausal women, gaining >5% of pre-diagnosis weight within two years of a breast cancer diagnosis was associated with increased all-cause mortality (HR = 5.87 (95% CI, 0.89–47.8)) [39]. Given that the effects of obesity on tumor biology described here are independent of menopausal status, it is clear that reconciling the effect of obesity on incident cancer in premenopausal and postmenopausal women must extend to other mechanisms without losing sight of the importance of obesity-mediated alterations in sex hormone metabolism.

## 5. Other Mechanisms Underlying the Effects of Obesity on Breast Carcinogenesis

A number of years ago, the seminal reviews of Calle and Kaaks [40] and more recently of Renehan [15] solidified the expansion of potential mechanisms beyond sex hormone metabolism to include deregulated insulin signaling, altered adipokine metabolism, and chronic subacute inflammation. We will briefly review the strengths and limitations associated with each mechanistic area before introducing how emerging concepts and recent breakthroughs in our understanding of carcinogenesis may reshape our current understanding of how obesity affects the development of breast cancer.

## 6. Deregulated Insulin Signaling in Obesity and Cancer

The co-existence of deregulated insulin signaling in conjunction with obesity has been recognized for decades, leading Astrup and Finer to coin the term “diabesity” [41]. BMI is positively correlated with circulating insulin and a related growth factor, insulin like growth factor-1 (IGF-1). That the positive association of insulin and IGF-1 with BMI would be procarcinogenic was proposed two decades ago [42,43]. It was suggested that hyperinsulinemia contributes to cancer development through direct effects of insulin on growth-promoting signaling, and indirectly through the effects of prolonged hyperinsulinemia that increase the availability of bioactive IGF-1 via the reduction of circulating levels of the proteins (IGF binding proteins) that normally bind IGF-1 and make it biologically unavailable.

Higher insulin and IGF-1 have the potential to create a pro-carcinogenic environment. Specifically, binding of insulin or IGF-1 to their cognate receptors activates a series of events including the phosphatidyl inositol 3 kinase (PI3K)/Akt/mammalian target of rapamycin (mTOR) signal transduction cascade. This pathway culminates in the activation of S6 kinase and releasing the inhibition of the eIF4e transcription factor. Cumulatively, these effects induce expression of proteins involved in cell cycle progression, including cyclin D1 and c-Myc [44,45,46,47,48,49,50]. Insulin signaling also prevents apoptosis via increased expression of anti-apoptotic proteins Bcl-2 and Bcl-XL, with concomitant suppression of pro-apoptotic protein Bax [44,45,46,47,48,49,50]. Consistent with this premise, risk for breast cancer is increased in association with higher circulating levels of insulin and IGF-1. Hyperinsulinemia as a product of insulin resistance is associated with increased breast cancer risk in postmenopausal women (comparison of highest to lowest quartile, HR = 1.46 (95% CI, 1.00–2.13)) [47]. Similarly, high IGF-1 is associated with increased breast cancer risk in both premenopausal women (comparison of highest to lowest quintile, odds ratio (OR) = 1.2 (95% CI, 1.0–1.5)) and postmenopausal women (OR = 1.33 (95% CI, 1.1–1.6)) [49].

Beyond breast cancer risk, insulin and IGF-1 stimulate cancer progression and invasion. High levels of insulin signaling promote tumor angiogenesis through upregulation of vascular endothelial growth factor (VEGF) and hypoxia inducible factor (HIF)-1α, stimulation of endothelial cell proliferation, and promotion of vascular tube formation [51]. High circulating insulin increases risk of breast cancer recurrence (comparison of highest to lowest quartile, HR = 2.0 (95% CI, 1.2–3.3)) [52]. It has also been reported that obese insulin-resistant individuals had elevated risk of cancer mortality (HR = 1.52) compared to obese insulin-sensitive individuals (HR = 1.04) [53].

Nonetheless, when this information is considered relative to the effects of obesity on cancer characteristics at the time of diagnosis, there are many inconsistencies; e.g., a direct effect of IGF-1 on premenopausal cancer *versus* the inverse association commonly reported. In the Cecchini study [16] of pre- and postmenopausal women with high Gail risk scores, a greater percentage of obese (5.9%) and overweight (2.3%) women were diabetic than lean women (1.4%). However, full multivariable adjustment for several variables including diabetes only slightly reduced hazard ratios in premenopausal women from the final multivariable assessment of 1.59 and 1.70 for overweight and obesity to 1.55 and 1.66, respectively [16]. As adjustment for diabetes did not substantially influence risk estimates, factors in addition to insulin signaling appear to be influencing breast cancer risk in overweight and obese populations.

Other concerns or inconsistencies relative to insulin signaling as a candidate mechanism driving the relationship between obesity and poor breast cancer outcomes include:

(1) The difficulty in measuring circulating levels of bioavailable IGF-1, as no validated assays are currently available;
(2) Uncertainty relative to the usefulness of reported levels of serum insulin, which are highly dependent on the state and duration of fasting, assay characteristics and genetic factors;
(3) Possible reporting bias for studies describing significant associations between either insulin or IGF-1 and cancer risk [54];
(4) Recognition that if insulin signaling is invoked as a candidate mechanism to explain adiposity and cancer risk, one would expect that exogenously administered insulin in patients with diabetes might be associated with increased cancer risk, but it is not [55,56];
(5) Concern that the extensive preclinical literature on supraphysiological levels of insulin on tumor development are not applicable to humans [57]; and
(6) Total levels of IGF1 in mice increase with increasing fatness across a wide range of weights, yet this is not observed in clinical populations, in whom total levels of IGF-1 increase only to a BMI of approximately 27 kg per m^2^, thereafter declining with increasing weight [58,59].

## 7. Chronic Inflammation as a Permissive Environment for Breast Carcinogenesis

As a compensatory mechanism for chronic positive energy balance, obesity is characterized by both adipocyte hyperplasia (differentiation of preadipocytes into mature adipocytes) and adipocyte hypertrophy (increased size of existing mature adipocytes). Compared to smaller adipocytes, hypertrophic adipocytes display increased rates of lipolysis, increased free fatty acid turnover from adipocytes, and increased risk of adipocyte death [60,61,62,63]. In a study of healthy men and women undergoing elective abdominal surgery, adipocytes with the largest diameter displayed increased expression of TNF-α, IL-6, colony stimulating factor (CSF)-1, and macrophage chemoattractant protein (MCP)-1, which recruits macrophages to dysfunctional adipocytes compared to adipocytes with the smallest diameter [64].

From a physiological perspective, inflammation is a necessary response to tissue injury associated with obesity induced adipocyte dysfunction. Expression of pro-inflammatory cytokines, including interleukin (IL)-1 α/β, IL-6, interferon (IFN)-γ, tumor necrosis factor (TNF)-α, and transforming growth factor (TGF)-α/β, is usually closely followed by the production of anti-inflammatory cytokines within the tissue, including IL-10, IL-13, and IL-14, under normal acute conditions (reviewed in [65]). However, this negative feedback loop is impaired in the pathological states of obesity and cancer, which both demonstrate chronic low-grade inflammation. At a molecular level, adipose tissue inflammation can impact tumor development by effects on pathways related to proliferation, suppression of apoptosis, and angiogenesis [66,67]. For example, IL-6 activates the signal transducer and activator of transcription (STAT)-3, which translocates to the nucleus and stimulates transcription of genes including cyclin D1 and c-myc [68]. STAT-3 stimulates expression of Bcl-XL and survivin in orthotopically-induced breast tumors in mice to prevent apoptosis [69]. TNF-α activates NF-κB, which in conjunction with STAT3 promotes angiogenesis through upregulation of hypoxia-inducible factor (HIF)-1α and vascular endothelial growth factor (VEGF) [67,68].

Epidemiologic data on the association of inflammatory factors with obesity and breast cancer are limited primarily to investigations of systemic biomarkers which have short half-lives and that are unlikely to reflect local, tissue-specific effects of obesity on inflammation. Of the biomarkers evaluated, C-reactive protein (CRP), an inflammation-associated, nonspecific biomarker is most commonly studied with the advantage that it has a longer half-life than other circulating biomarkers of inflammation [70]. Circulating CRP levels were positively correlated with BMI (regression coefficient = 1.35 (95% CI, 1.25–1.44)); fat mass (regression coefficient = 2.52 (95% CI, 2.33–2.70)); and waist circumference (regression coefficient = 3.27 (95% CI, 3.03–3.52)) in healthy women [71]. However, CRP generally has not been found to be statistically significantly associated with breast cancer risk [70,72,73,74,75,76,77], although most studies evaluated a relatively small number of breast cancer cases and most included both pre- and postmenopausal breast cancers without regard for molecular subtype of breast cancer. A nested case-control study conducted in the Multiethnic Cohort that included *N* = 706 postmenopausal breast cancer cases and *N* = 706 matched-controls, reported a positive, albeit non-linear, association between circulating CRP and breast cancer risk, even after adjustment for BMI and other breast cancer risk factors [78]. A prospective analysis in the E3N cohort in France did not report a statistically significant association between CRP levels and breast cancer risk overall, though there was evidence for a statistical interaction with BMI, such that higher CRP levels were positively associated with breast cancer incidence among overweight and obese women [72].

In women with primary breast cancer diagnosis recruited to the Health, Eating, Activity, and Lifestyle (HEAL) Study, circulating levels of inflammatory biomarkers serum amyloid A (SAA) and C-reactive protein were associated with reduced disease-free survival (comparison of highest to lowest tertiles, SAA: HR = 2.91 (95% CI, 1.61–5.26); CRP: HR = 2.05 (95% CI, 1.14–3.69)) [79]. In a separate study by Sheen-Chen *et al.*, women with primary breast tumors had 1.5-fold elevations in circulating TNF-α compared to cancer-free women, and TNF-α levels were positively correlated with increasing tumor stage and tumor size [80]. Most recently, higher circulating CRP levels were associated with increased incidence of breast cancer among women not using hormone therapy among WHI participants. Specifically, the breast cancer incidence rate in these women was two-fold greater among those in the highest two quartiles compared with those in the lowest quartile of CRP, even after controlling for estradiol, insulin, BMI and established breast cancer risk factors. Nonetheless, a review of epidemiological studies of biomarkers and subsequent cancer risk has raised concerns about biased reporting in studies of inflammation biomarkers [54].

## 8. Altered Adipose Tissue Endocrine Function in Obesity Has Implications on Breast Cancer

While previously thought of as a passive storage depot for excess energy, adipose tissue is now recognized as a powerful endocrine organ with far-reaching effects on multiple disease processes. These actions are exerted via adipose-derived cytokines, known as adipokines. Of the more than 40 adipokines that have been identified thus far, leptin and adiponectin are among the most abundant in human serum [81]. Whereas circulating leptin is positively correlated with adiposity [82,83], adiponectin is generally lower in obese compared to lean individuals [81].

### 8.1. Leptin and Breast Cancer

Data on the association of leptin with breast cancer risk are mixed [84,85,86,87,88]. For example, Wu *et al.* reported that levels of circulating leptin were associated with increased breast cancer risk in both premenopausal women (HR = 1.40 (95% CI, 0.83–2.38); comparison of highest to lowest tertile) and postmenopausal women (HR = 1.69 (95% CI, 0.95–3.06)) [88]. When waist circumference was included as a covariate, breast cancer risk associated with circulating leptin was further increased in premenopausal women (comparison for highest to lowest quartile, HR = 1.99 (95% CI, 1.06–3.39)) and postmenopausal women (comparison for highest to lowest quartile, HR = 3.25 (95% CI, 1.53–6.91)) [88]. In a study of 35 primary breast cancers, 75% of tumors expressed leptin and 80% of tumors expressed the leptin receptor [89]. Expression of the leptin receptor was correlated with expression of ER and tumor size [89]. Normal breast tissue adjacent to cancerous tissue displayed increased leptin expression in 60% of the cases evaluated; in contrast, leptin was undetectable in the breasts of cancer-free women [90]. Breast tumor expression of leptin receptor in conjunction with elevated circulating leptin is associated with poor prognosis and tumor metastasis [91,92]. Two clinical reports fail to link circulating leptin with breast cancer recurrence [93,94], although another has shown leptin to be associated with distant recurrence and death even when statistical models were adjusted for BMI and body weight [95].

At a molecular level, leptin displays considerable cross-talk with estrogen signaling [92]. Leptin has been shown to stimulate proliferation of breast cancer cell lines *in vitro* [96], to stimulate tumor growth in nude mice via activation of the extracellular signal-related kinase (ERK) [97], and to stimulate growth of carcinogen-induced rat tumors [98]. Leptin also may inhibit apoptosis via upregulation of the anti-apoptotic protein survivin [99,100]. In terms of a potential causal role of leptin as a driver of breast carcinogenesis, preclinical data show that leptin regulates JAK2/STAT3 and inflammatory cytokine related signaling, which is altered by obesity and reregulated via weight loss [101,102]. Plasma leptin data have to be interpreted with caution since leptin and the two main isoforms of its receptor are expressed in 84% of breast cancers, suggesting that cells within tumors can respond to leptin via autocrine as well as paracrine and endocrine pathways [103].

### 8.2. Adiponectin and Breast Cancer

Adiponectin was inversely associated with breast cancer incidence in some prior prospective investigations [86,104] but not in other studies [84,85], although three recent meta-analyses that included both prospective cohort and case-control studies reported an inverse relationship between adiponectin levels and breast cancer risk [105,106,107]. Macis *et al.* reported that circulating adiponectin is associated with reduced breast cancer risk in both premenopausal women (summary RR = 0.72 (95% CI, 0.30–1.72)) and postmenopausal women (summary RR = 0.80 (0.63–1.01)) [106], though findings reported by others are mixed (*i.e.*, [108,109,110]).

Adiponectin exerts systemic insulin-sensitizing effects through increased glucose uptake and increased β-oxidation in muscle [111]. Likewise, adiponectin inhibits liver gluconeogenesis via activation of AMP-activated protein kinase [111]. Adiponectin also suppresses inflammation, as overexpression of adiponectin in *lep*^ob^/*lep*^ob^ mice was associated with reduced macrophage infiltration of fat, corresponding to an anti-inflammatory phenotype. Administration of adiponectin induced a switch from M1-type macrophages (pro-inflammatory) to M2-type macrophages (anti-inflammatory) in *lep*^ob^/*lep*^ob^ obese mice (reviewed in [112]). There are three major oligomeric forms of adiponectin: a low-molecular-weight (LMW) trimer, a middle-molecular-weight (MMW) hexamer and a high-molecular-weight (HMW) multimer. LMW oligomers are the predominant form in the circulation, whereas the majority of intracellular adiponectin consists of HMW multimers. The ratio of HMW to LMW is critical to insulin sensitivity. Both adiponectin receptors (ADIPOR1 and ADIPOR2) may exist in tumor cells, but more studies are needed to characterize these subtypes and their functions.

### 8.3. Other Considerations

The biological effects of adipokines such as leptin and adiponectin depend not only on relative circulating concentrations but also the form present in tissue and the tissue-specific expression of receptor subtypes. Moreover, studies in fatless A-Zip/F1 mice, which have undetectable adipokine levels in the circulation but display accelerated tumor formation, suggest that adipocyte derived adipokines are not essential for tumor development [113]. Given their opposing impact on breast cancer risk, some have proposed that the adiponectin: leptin ratio, rather than levels of individual adipokines, may be more informative for evaluating breast cancer risk profile [114,115]. However, the utility and biological relevance of expressing circulating concentrations of leptin and adiponectin as ratios in clinical populations has recently been questioned, particularly since these proteins do not directly interact and each is involved in cross talk with other signaling pathways [116].

## 9. The Multiple-Hits Hypothesis for Carcinogenesis

The cancer genome atlas project has created a wealth of information which has informed current understanding of the mechanisms that underlie the development of cancer and that potentially impact the resolution of the obesity-breast cancer conundrum [117]. There are two concepts that merit particular attention.

Tomasetti and Vogelstein recently advanced the hypothesis that a primary driver of the development of cancer is the occurrence of mutations during DNA replication [118]. The rate of mutation during cell division is remarkably similar in all tissues [119]. While controversial, this theory posits that cancer risk in a tissue is determined in large part by the number of cell doublings that the stem cells in that tissue normally undergo [120,121,122]. This concept was evaluated for a large number of organ sites and was found to be remarkably predictive of cancer rates in the United States. Moreover, even when exposures to environmental carcinogens and hereditary factors were taken into account, the mutations in stem cells occurring during DNA replication were still estimated to be a major source of the genomic changes leading to the development of cancer. While not examined for breast cancer because of the limited information available on mammary stem cells, the implications of this hypothesis are important to consider.

Development of the mammary gland is regulated at three critical stages: embryogenesis, puberty, and pregnancy, and is further influenced by lactation and involution [123]. The hormonal milieu and environmental exposures, including changes in height, weight, and adiposity, may regulate the size of the breast stem cell pool and its mitotic activity, and in so doing influence the carcinogenic process [123]. The mammary gland undergoes the majority of its development postnatally with significant changes in stem cell populations. Some stem cell populations increase to support mammary gland development, such as during pregnancy, and are later eliminated from the tissue [124]. But the dynamics of stem cell number and mitotic activity, as well as the impact of environmental exposures such as obesity, on the size and turnover of stem cell populations relative to cancer cell initiation and the subsequent emergence of cancers during premenopausal and postmenopausal life stages, is poorly understood.

Likewise, it is currently unknown how the molecular subtypes of breast cancer relate to specific stem cell populations and the cell populations they generate, e.g., basal-derived stem cells *versus* luminal-derived stem cells [125]. Of equal interest are data indicating the importance of steroid hormones, particularly progesterone [126,127,128,129], on the induction and regulation of mammary stem cells, as well as insulin and related growth factors [130] and adipokines such as leptin and adiponectin [131]. Many of the apparent contradictions identified in preceding sections may be reconciled as the role of stem cell compartments and their rates of cell division in the mammary gland are elucidated.

At this point, it must be reiterated that cancer is not a continuous process, but rather that it occurs in steps [132]. The stepwise model indicates that a specific sequence of acquired genomic events over many years characterize the transition from normal epithelium to invasive carcinoma and that specific driver events, acquired in a particular order, enable cells to progress from benign growth to an invasive phenotype. The stepwise nature of cancer occurs in as few as three steps defined as initiation (breakthrough), promotion (expansion), and progression (invasion). It cannot be presumed that obesity exerts equivalent effects on all steps in the process. Thus, it is important to determine which steps need to be examined relative to incident breast cancer risk *versus* disease progression based on menopausal status and molecular subtypes of breast cancer, and how the four widely embraced mechanisms discussed above may be operating, if at all, in affecting the course of the disease in the presence or absence of obesity.

## 10. Effects of Obesity on Other Mechanisms of Carcinogenesis: Relevance of the Cancer Hallmarks

While most of the discussion about obesity and cancer presented in preceding sections is based on aggregated data and statistical analyses, it is widely recognized that the field is moving beyond this approach towards understanding each individual, *i.e.*, personalized medicine. For an individual, risk can be affected in numerous positive and negative ways, by intrinsic genetic, epigenetic, and environmental influences [122,133]. Beyond classical genetic regulation, cancer hallmarks are subject to epigenetic regulation by obesity-related mechanisms [66]. Obesity was recently associated with hypermethylation of gene loci involved in inflammation, insulin signaling, and leptin signaling in normal breast tissue from cancer-free women [134]. In addition, obesity was associated with hypermethylation of gene loci involved in regulation of immune response, proliferation, and DNA repair in breast cancer patients with ER+ tumors [135]. MicroRNAs (miR) represent another epigenetic mechanism for the regulation of cancer hallmarks by obesity-related mechanisms. Obese individuals display altered microRNA expression profiles [136]. Changes in activity of the miR-processing machinery Dicer have been observed in obesity [136] and in breast cancer [137]. MiRs can have tumor suppressing or tumor promoting effects, which are manifested through regulation of replicative immortality, angiogenesis, inflammation, proliferation, and the epithelial to mesenchymal transition (reviewed in [138,139]).

Obesity has been reported to reduce survival among women with ER+ breast tumors (progression-free survival HR = 1.95 (95% CI, 1.02–3.75)) [140]. Likewise, within this study, obesity negatively influenced cancer-free survival in mice carrying an activating mutation in transforming growth factor (TGF)α under control of the mouse mammary tumor virus (MMTV). To provide insight into molecular mechanisms that may be associated with the worse breast cancer outcomes, this study profiled breast tumors from women and mice using microarrays. The data revealed that obesity was associated with altered expression of 85 genes in both mice and humans. When these 85 genes were ranked by average *Z*-score, the most common cancer hallmark to which these 85 genes mapped was sustained proliferation (Top 5 genes: *DUOX1* (dual oxidase 1), *SURF1* (surfeit 1), *CYCS* (cytochrome c (somatic)), *GNPDA1* (glucosamine-6-phosphate deaminase 1), *PHKA1* (phosphorylase kinase, α1)). The second most common hallmark to which these 85 genes mapped was evasion of apoptosis (Top 5 genes: *LEP* (leptin), *PDIA5* (protein disulfide isomerase family A, member 5), *ALDH5A1* (aldehyde dehydrogenase 5 family, member A1), *SDHD* (succinate dehydrogenase complex, subunit D), *PHKG2* (phosphorylase kinase, gamma 2)). These data suggest that the balance between proliferation and apoptosis is an important mechanism whereby the promotional effects of obesity on the carcinogenic process in the breast are mediated, a finding consistent with a recent report on the effect of obesity on breast cancer growth rates in a premenopausal model in the rat [141].

With all the new information that is emerging, it is easy to lose sight of the trees in the forest: namely, that the development of cancer does not occur if clonal expansion of cancer initiated cells does not result in cell mass accumulation due to the selective growth advantage conferred by driver mutations [117]. While the mechanism through which cell accumulation occurs is frequently attributed to increased cell proliferation, that view is incomplete in that cell accumulation only occurs if an imbalance exists between cell birth and cell death; the magnitude of the shift in equilibrium is estimated to be 0.4% per acquired driver mutation [117,142], an observation consistent with the hallmarks of cancer [66,143]. As summarized extensively in Steel’s classic work on tumor growth kinetics [144,145,146], and as applied to the process of clonal expansion [117,142,147], the accumulation of tumor cell mass is the net result of the growth fraction of the cells within the tumor, the rate of their transit through the cell cycle, and the rate of cell loss, with necrosis and apoptosis being important potential contributors to cell loss. A third cellular process that is intimately associated with cell proliferation and cell death within emerging pathologies that directly relates to energy availability is angiogenesis. Angiogenesis is a critical regulator of tumor growth, because as the growth of a tumor expands beyond the limits of diffusion, new blood vessel formation is required to supply cells with oxygen and nutrients and to remove metabolic byproducts. Since the timeframe for the development of obesity and cancer is prolonged and the imbalance between cell proliferation and cell death is small at any snapshot in time [142], evaluation of the impact of obesity per se on these cancer hallmarks is problematic, even though the linkage is obligatory.

## 11. Preclinical Models: What Tools Are Available?

Clearly, the relationship between obesity and breast cancer is extremely complex. Preclinical models are valuable tools that can be used to deconstruct the biology of this problem in a stepwise manner by interrogating specific pieces of the puzzle. However, models must be selected carefully based on the clinical question under investigation, such that the biology involved in humans is adequately represented in the chosen preclinical model.

## 12. Rodent Models of Obesity and Breast Cancer

### 12.1. Monogenic Models of Obesity

Heritable cases of obesity in humans typically display mutations associated with leptin deficiency, truncated leptin receptor, pro-opiomelanocortin (POMC) deficiency, melanocortin-4 receptor deficiency, and prohormone convertase (PC)1 deficiency [148]. Thus, models are available in mice and/or rats include the *lep*^ob^/*lep*^ob^ and *lepr*^db^/*lepr*^db^ strains, which possess mutations in the genes that encode leptin and the leptin receptor, respectively. These models have provided important mechanistic information relative to obesity, although their use in resolving the obesity-breast cancer conundrum must be carefully considered given the above-cited evidence that leptin plays a role in that relationship.

### 12.2. Polygenic Mouse Models of Obesity

The majority of obesity cases in humans are attributable to multiple factors including polygenic susceptibility [149]. To develop preclinical models that are more representative of the polygenic nature of most human obesity, Cleary *et al.* has reported identification of subpopulations of FVB mice with polygenic predisposition to obesity and such populations already exist in the B6 mouse strain [150,151,152]. This is important since most transgenic and knockout cancer models in mice are developed in these strains. These models are referred to as diet-induced obesity; mice are fed high fat diets containing 45%–60% kcal from fat, which is higher than the 30%–35% routinely consumed by most humans. As controls for diet-induced obese mice, non-obese mice are frequently fed a low fat diet containing 10%–11% kcal from fat. A limitation of this approach is that it adds differences in dietary composition as potentially confounding variables in those investigations [153].

### 12.3. Polygenic Rat Models of Obesity

In contrast to mice, most rat strains are resistant to diet-induced obesity (DIO) in the short term; however, MacLean *et al.* have characterized subpopulations of Wistar rats with acute sensitivity or resistance to diet-induced excess weight gain [154,155,156,157]. These studies utilized 45% kcal from fat. In 1997, a novel model for the study of diet-induced obesity was introduced by Barry Levin of Veterans Affairs Medical Center in East Orange, NJ, USA. This model utilizes two strains of Sprague Dawley rats selectively bred for >20 generations for hereditary resistance (DR) or susceptibility (DIO, or DS, as used herein) to diet-induced obesity when fed diet containing ~32% kcal as fat. This model has been extensively characterized by Levin’s group (e.g., [158,159,160]). When fed the 32% fat diet, DS rats rapidly gain excess weight and have expanded peripheral and visceral fat depots by three months of age. DS rats display multiple metabolic abnormalities, including hyperlipidemia (total cholesterol and triglycerides) by two months of age, hyperleptinemia by three months of age, and pronounced fat infiltration of the liver by six months of age [159]. DS rats display prediabetic measures of glucose homeostasis, including hyperinsulinemia and worsened oral glucose tolerance by two months of age, insulin resistance by three months of age, and eventual reduced pancreatic insulin secretion by nine months of age, though rats do not fully progress to diabetes up to two years of age [159]. The Levin DS rats display many of the same comorbidities as manifested in the human obese condition, and do so when both lean (DR) and obese (DS) strains are fed the same diet. The macronutrient composition of the Levin diet is highly similar to that of the average American woman.

### 12.4. Mammary Carcinogenesis

Mouse and rats models exist for breast cancer and this topic has been reviewed extensively [161,162,163]. Luminal molecular subtypes of breast cancer are the most commonly observed in clinical populations. Luminal breast cancers derive their name from similar protein expression profiles as non-neoplastic cells which line the lumen of the breast duct. In the human breast, approximately 10%–30% of normal luminal epithelial cells express sex hormone receptors, including the estrogen receptor (ER) and the progesterone receptor (PR) [164,165]. As noted above, obesity predominantly increases risk of luminal (ER/PR+) breast cancer subtypes. Rat models are best suited to study sex steroid receptor positive breast cancer. Breast cancer is induced in the rat via carcinogen treatment and the tumors that occur have high histological and morphological similarity to human breast cancer and are predominantly ER/PR positive [166]. By combining the Levin strains of rats with our laboratory’s rapid emergence model of mammary carcinogenesis, we have developed a novel preclinical model with high relevance to clinical populations [141,167].

In contrast, most mammary tumors that arise in mice are hormone receptor negative (ER/PR-) tumors, although gene targeted knockouts (e.g., STAT1) are being reported that overcome this limitation. In general, mouse models for breast cancer are better suited for studying how obesity affects hormone receptor negative and Her2/neu overexpressing molecular subtype of the disease, as well as hormone refractory luminal breast cancer. On the other hand, the rat mammary gland is more similar to the human mammary gland in terms of fibrous and connective tissue content [168]. However, depending on the question being addressed, it is likely that a combination of models will provide the strongest approach to deconstructing the obesity-breast cancer conundrum. In this context, mouse models are of particular value due to the ability to modify (knock-in or knock-out) expression of specified genes in a tissue-specific manner [163].

## 13. Conclusions

We argue that obesity is a complex, multifaceted condition and that active avoidance of excessive weight for height, irrespective of menopausal status, has a direct and consistent bearing on the number of women who die from this disease. Thus, it is critical to fully investigate how obesity impacts the carcinogenic process in the breast. This effort will be facilitated via the identification of pivotal mechanistic questions and the informed use of preclinical models to assist with the deconstruction of the complex interrelationships that underlie the obesity-breast cancer conundrum. For success, it is essential to move the analysis of the effects of obesity on breast carcinogenesis from the population-based framework to a level consistent with the progress being made in oncology via precision medicine. In this regard, prevention of breast cancer via weight control must consider the emerging approaches to prevention as recently summarized [122,133] and the new insights to cancer causality being ushered in by the understanding of the cancer genome landscapes [117,118,132].

## Abbreviations

The following abbreviations are used in this manuscript: BMI, body mass index; CI, confidence interval; ER, estrogen receptor; HR, hazard ratio; OR, odds ratio; PR, progesterone receptor; RR, relative risk.
